# Complex Regional Pain Syndrome

**DOI:** 10.1055/s-0044-1779331

**Published:** 2024-04-22

**Authors:** Giana Silveira Giostri, Camila Deneka Arantes Souza

**Affiliations:** 1 Serviço de Ensino e Treinamento em Cirurgia da Mão, Hospital Cajuru/PUCPR, Curitiba, PR, Brasil; 2 Hospital Pequeno Príncipe, Curitiba, PR, Brasil; 3 Hospital Evangélico Mackenzie, Curitiba, PR, Brasil

**Keywords:** chronic pain, complex regional pain syndrome, hyperalgesia

## Abstract

Complex Regional Pain Syndrome is characterized by regional pain that is disproportionate to the triggering event, with no distribution to dermatomes, a tendency towards chronicity, and dysfunction. This narrative review proposes an update of criteria for diagnosis and management of the syndrome, providing information on epidemiology, etiology, and pathophysiology. We base our information on systematic and narrative reviews, as well as guidelines published in recent years, aiming to facilitate diagnostic suspicion and provide a broad overview of therapeutic possibilities.

## Introduction

Complex Regional Pain Syndrome (CRPS) is a syndrome that represents a great challenge to diagnosis and especially management, made difficult by numerous interventions with little scientific basis and evidence. It is characterized by chronicity and progressive worsening of symptoms that can lead to significant functional disability in patients. The diagnosis is based on a careful history analysis and, more importantly, on a thorough physical examination. 1 In general, it differs from other conditions that determine chronic pain by presenting prominent autonomic and inflammatory changes in a segment or region, without following dermatomes. 2 3 More common in upper than lower limbs, it is more frequently unilateral. 4 Laboratory studies are irrelevant, although useful for differential diagnosis in certain circumstances. Radiographs may show signs of regional osteopenia. Early diagnosis and treatment are essential to minimize permanent loss of limb function.

From the beginning of the 1990s, the term Complex Regional Pain Syndrome was adopted from what previously received terms such as reflex sympathetic dystrophy. 5 The International Association for the Study of Pain (IASP) began to establish parameters, known as the Budapest criteria, which seek to assist in the diagnosis and categorization of the clinical picture ( Chart 1 ). 6 These parameters have been validated in guidelines seeking greater specificity, since useful clinical criteria may differ not only between patients, but over time in the same individual.

**Chart 1:** Pain criteria for Complex Regional Pain Syndrome (CRPS) revised and adopted by the IASP (International Association for the Study of Pain) in 2012

**Characteristics:**
CRPS is a syndrome characterized by a continuing regional pain (spontaneous and/or evoked) that is seemingly disproportionate in time or degree to the usual course of any known trauma or other lesion. The pain is regional (not in specific nerve territory or dermatome) and usually has a distal predominance of abnormal sensory, motor, sudomotor, vasomotor, and/or trophic findings. The syndrome shows variable progression over time.
**Clinical Criteria for Diagnosis of CRPS**
1. Continuing pain disproportionate to the inciting event 2. They should report at least one symptom in three of the four following categories: Sensory : Reports of hyperalgesia and/or allodynia, Vasomotor : Reports of temperature asymmetry and/or skin color changes and/or skin color asymmetry, Sudomotor/edema : Reports of edema and/or sweating changes and/or sweating asymmetry, Motor/trophic : Reports of decreased range of motion and/or motor dysfunction (weakness, tremor, dystonia) and/or trophic changes (hair, skin, nails) 3. they must display at least one sign* at the time of evaluation in two or more of the following categories: Sensory : Evidence of hyperalgesia (to pinprick) and/or allodynia (to light touch or deep somatic pressure), Vasomotor : Evidence of temperature asymmetry and/or skin color changes and/or asymmetry, Sudomotor/edema : Edema and/or sweating changes and/or sweating asymmetry, Motor/trophic : Evidence of decreased range of motion and/or motor dysfunction (weakness, tremor, dystonia) and/or trophic changes (hair, skin, nails). * A sign is only counted if it was observed at the time of diagnosis. 4. There is no other diagnosis that better explains the signs and symptoms

Source: Harden RN, McCabe CS, Goebel A, et al. Complex regional pain syndrome: practical diagnostic and treatment guidelines, 5th edition. Pain Med 2022;23(Suppl 1):S1-S53. 6

## Types of CRPS

Classically, CRPS is presented in three subtypes. In type I, there is no specific involvement of the peripheral nerve, evidenced on clinical or electrophysiological examination, a relatively limited syndrome in which vasomotor signs predominate. In type II, previously called causalgia, there is involvement of identifiable nerve damage that determines neuropathic pain, a relatively limited syndrome in which neuropathic pain/sensory abnormalities predominate. Some authors refer to a third type that partially meets the criteria, however, they are not specified in the other two types, type III is defined as a very florid CRPS, although signs of motor/trophic changes were observed in all three groups, they doubled in frequency among Subgroup 3 patients. 3 4 7 Apart from the previously mentioned subtypes I, II, and III, there is reference to clinical differentiation of the syndrome into "hot" and "cold" patterns, respectively characterized by a hot, swollen, dry, and reddish extremity, and another by a cold extremity, less swollen, with sweating and a bluish hue. Harden et al. reported that the more common pattern is the "hot" pattern, which has a shorter duration of the syndrome compared to the "cold" pattern. 7

## Epidemiology

CRPS has a frequency of 5.46-26.2 per 100,000 people per year. 6 8 Women are three times more affected than men. The most prevalent age ranges between 46 and 53 years. Upper limbs are more involved than the lower limbs. Shim et al. 5 reported that there will be a greater risk of developing CRPS in women who suffer high-energy trauma to the upper limb. Bruehl 2 reports different incidence rates for types I and II of the syndrome, respectively 5.46 per 100 thousand people per year and 0.82 per 100 thousand people per year.

## Etiology and Pathophysiology

Fractures are referred to as the main precipitating factor for CRPS in the upper and lower limbs, with fractures of the radius and ulna at their distal extremity being the region most affected by the syndrome. 1 CRPS is more likely to occur after distal radius fractures in patients treated with tight casts or after a nociceptive or neuropathic event such as median nerve compression, hyperdistraction, distal radioulnar joint instability, or ulna fracture. 1 Other traumas, surgeries and even inflammatory conditions can be manifesting factors. Urits et al., 3 mentioned that in 7% of cases, type I syndrome develops usually within 8 weeks following the traumatic event. It is important to note the persistence of pain and edema after the first weeks of trauma as signs of the onset of the syndrome. There is a report of an incidence slightly higher than 8% in patients undergoing carpal tunnel release. 3 However, in a recent systematic review, which searched for available evidence on the occurrence of CRPS after surgical release of the carpal tunnel, our group found an incidence of 2 to 5%. And the following are listed as risk factors for the onset of the syndrome after surgery: female sex, age from the fifth decade, surgery on the dominant hand, tourniquet time and use of immobilization after surgical procedure. 8

The pathophysiology of CRPS is uncertain. The exact mechanism that determines the onset and maintenance of pain remains unknown. 3 Theories tend to focus on why pain persists rather than how it arises. It is hypothesized that neuropeptides linked to the gene of chemical modulators, such as calcitonin, incite neurogenic inflammation and catecholamine sensitization. The persistent nature of pain in CRPS appears to be connected to autonomic characteristics of the sympathetic nervous system, such as upregulation of adrenoceptors and decreased density of cutaneous nerve fibers. 9 Pathological pain appears to come from changes in sensory and motor processing, that is, motor function, sensory feedback, body representation, proprioception and sensorimotor integration. However, there is very limited evidence that changes in this processing would actually lead to chronic pain. 10 The multifactorial theory is more accepted and includes aspects that interfere from the moment of the offending event, such as nervous system sensitization, autonomic dysfunction, inflammatory changes, in addition to a possible relationship with psychological factors and genetic predisposition. 5 Also, smoking was identified as a significant risk factor for the development of CRPS. 7

Dysfunctions caused by CRPS may include loss of normal arteriovenous flow mechanisms and definitive changes in central neurological responses, resulting in segmental ischemia and cell death. 10 These, in turn, cause pain, edema, joint stiffness and atrophy of the segment. In summary, complex regional pain syndrome essentially brings exaggeration or abnormal prolongation of pathophysiological events expected after a certain traumatic episode, altering perception and subjecting the individual to a complex interaction of physiological and psychological factors. 10

## Diagnosis

The suspicion of the development of CRPS begins with the appearance of exacerbated pain, normally not expected after trauma or surgery. Other factors must be considered, such as unexpected maintenance of edema, slower than usual recovery, sleep disorders and lack of pain improvement with common analgesics. 11 The diagnosis can be aided by using the Budapest criteria ( Chart 1 ). 6

It is distinguished from other painful syndromes by the presence of autonomic dysfunction, persistent regional inflammatory changes, and the absence of distribution following dermatomes. 5 Patients describe the pain as tearing or burning, often exacerbated by cold. In addition to difficulty sleeping, they also report anxiety and restlessness. Commonly found on physical examination are allodynia (painful response to normally non-painful stimuli), hyperalgesia and hyperpathy associated with edema. Trophic changes resulting from autonomic dysfunction may be present, such as abnormal sweating, changes in temperature and skin texture. The involved extremity is identified as “hot” and edematous or “cold” and atrophic. Over time, pain and swelling lead to joint stiffness, especially in the fingers, wrists and shoulders. 10

Children and adolescents can be affected by CRPS. It is more common in the lower limbs of adolescent girls, around 12 years of age, who have had a traumatic event in the ankle and foot region. 1 The clinical picture includes pain, autonomic changes and heightened sensitivity to painful and even non-habitually painful stimuli (allodynia). As in adults, it can lead to motor disorders and trophic changes. The prognosis tends to be better compared to the occurrence in adults. 12

In the face of intense pain that tends to become chronic, it is essential to rule out other pathologies in the absence of objective physical findings, such as trophic changes, edema and osteopenia. Pain and disability linked to CRPS point to psychological comorbidities that create a vicious cycle of pain, isolation and depression. 4

Piñal (Enzine, 2019), in his true outburst published in the IFSSH (International Federation of Societies for Surgery of the Hand) magazine on the topic, casts doubt on the existence of types of the syndrome and exposes the controversy in diagnosis and treatment. It refers to the theory of stimulus amplification in a nerve that is being “irritated”, even without an established mechanical injury, which would result in more comprehensive symptoms in the affected limb. The author recently published a study of 53 patients, on average 55 years of age, previously diagnosed as having CRPS, who presented atypical symptoms and signs of pain and dysesthesia in the upper limb. He emphasized the inability of individuals to completely flex their fingers (grip), and demonstrated improvement in symptoms after releasing the transverse carpal ligament. Only 6 of the 53 patients maintained their condition prior to surgery, assessed with a visual analogue pain scale and the DASH questionnaire (Disabilities of the Arm, Shoulder and Hand Questionnaire). 13 In this line of reasoning, Chang et al. 4 discussed the labeling psychosocial effects of syndromes. They started from the premise that CRPS can present a set of inconsistent and vague symptoms, easily addressed in the context of other diagnoses. They cited examples of articles that confirmed high percentages of the syndrome after a certain surgical procedure without patients even completing signs and symptoms such as those mentioned in the criteria presented in the table. 2 They highlighted the importance of a thorough clinical examination to identify possible sources of pain and avoid incorrect procedures that could have disastrous consequences for the patient.

Complementary imaging and laboratory tests will generally be unchanged and will be of little help in establishing the diagnosis of CRPS. Two weeks or more after the onset of the condition, radiographs may show signs of osteopenia and bone resorption, mainly subchondral and periarticular. It is estimated that up to 30% of patients will not present radiographic abnormalities at the time of diagnosis of the syndrome. 10 Although thermography, scintigraphy, electroneuromyography and ultrasound can demonstrate changes in the presence of CRPS, ultimately, there is no scientific evidence for their indiscriminate use in diagnosing the syndrome. 5

## Treatment

The difficulty in proposing treatment in CRPS goes in the same direction as establishing its diagnosis. Numerous therapies are suggested in isolated case series or in comparisons, narrative reviews, guidelines and data grouped in systematic reviews and meta-analysis attempts. In a simple search on one of the scientific search platforms, it is possible to capture more than four thousand articles published between the years 1945 and 2022, the majority being after the year 2000. The first publications are found with the names of causalgia, reflex pain, dystrophy sympathetic reflex, Sudeck's dystrophy, which express the same difficulties found in more recent studies, that is, the discussion in establishing diagnosis and management. This leads us to reflect, we are studying the cause of this supposed syndrome, trying to discover its prevention or exposing countless treatment options for poorly recognized or misdiagnosed symptoms.

Patients with type 2 CRPS have an identifiable or documented peripheral nerve injury. Surgical interventions that correct nerve damage or protect the nerve can decrease the incidence of nociceptive foci, lessen symptoms, and improve function. Similarly, patients with type 1 CRPS may have a non-neural or mechanical nociceptive focus, which, if corrected, will facilitate extremity recovery. 1

The use of numerous drugs, neuraxial therapy, spinal cord and dorsal ganglion branch stimulation, intravenous regional and peripheral sympathetic blockade, adjuvant therapy, among others, are mentioned in the treatment of CRPS alone or together. 6 14 To present the current CRPS treatment options, we based ourselves on systematic reviews, narratives and guidelines from recent years. We seek to organize them into topics to facilitate understanding given the plethora of possibilities. However, studies suggest that high-quality randomized controlled trials are still needed to make solid recommendations regarding the treatment of CRPS. 1

### Drugs

- Bisphosphonates
They can be administered orally or intravenously; seek to inhibit bone reabsorption that accompanies CRPS. Research states that bisphosphonates have clear benefits in the treatment of the syndrome. 14 15 There are reports of significant improvement in allodynia, hyperalgia, edema and joint mobility. Pamidronate infusions are well tolerated and easy to administer. Several patients have reported minor infusion site reactions and mild symptoms that resolved within 6 to 24 hours after administration. The mechanism of the analgesic effect of bisphosphonates remains speculative. They are potent inhibitors of osteoclastic activity and may play a role in modifying inflammatory cytokines (such as interleukin-1) and other systemic factors (such as prostaglandin E2) involved in the sensitization of pain nociceptors and low-threshold mechanoreceptors. 15
- Corticosteroids
Christensen et al. 16 compared oral prednisone and placebo in the treatment of CRPS and achieved improvement in pain, edema, palmar sweat, pinching ability and fingering with the use of corticosteroids. These mediate the inhibition of inflammation and analgesia. Also, other authors reported better control of pain and edema with the use of oral prednisone when compared to non-hormonal anti-inflammatory drugs. 17
- Calcitonin
When compared to placebo or other therapies, such as physical therapy, it showed insignificant benefits in the treatment of CRPS. 14 Believing that its action would determine inhibition of bone resorption and analgesia mediated by beta endorphin.
- Non-steroidal anti-inflammatory drugs
Breuer et al. 18 found no significant difference in terms of improvement in pain and edema in patients diagnosed with the syndrome, when comparing the use of intravenous parecoxib and saline solution. Its use was increased due to its function of promoting COX2 inhibition, tending to reduce peripheral sensitization and normalize the local pressure pain threshold.
- Vitamin C
The systematic review carried out by Giusta et al., 19 evaluated the effectiveness of vitamin C in preventing type I CRPS in fractures or surgeries of the upper and lower extremities. Five of the six studies analyzed were in favor of the prophylactic use of a daily dose of 500-1000 mg of vitamin C for 45-50 days after orthopedic or traumatological care to prevent CRPS type I. Only one study did not find any benefit in vitamin C supplementation compared to placebo in the prevention of CRPS. Therefore, in this analysis of the literature, it is suggested that daily supplementation of 500-1000 mg of VC can reduce the appearance of type I CRPS in upper/lower extremity trauma and orthopedic surgery.
- N-Methyl-D-Aspartate (NMDA) receptor antagonist
Due to its action on peripheral and central sensitization mechanisms, specifically on the neuronal sensitization cascade, which leads to the activation and release of substances in the central nervous system (CNS) such as glutamate, increasing the efficiency of synaptic transmission of the pain signal. However, some articles reported no significant improvement with the use of NMDA antagonists or placebo. 18 20 21
Ketamine acts in the CNS as a non-competitive receptor blocker. Authors report a reduction in allodynia/hyperalgia with topical use of ketamine compared to placebo. 22 Good results were also reported with intravenous use. Attention should be paid to the short duration of intravenous treatment, avoiding undesirable symptoms such as nausea, vomiting and psychomimetic effects. 23 24
- Botulinum toxin A
As botulinum toxin tends to decrease peripheral sensitization and central pain perception by inhibiting both superficial and deep pain neurotransmission and blocking retrograde axonal transport, its action was evaluated in the treatment of CRPS. However, there is reference to intense pain during the application of the toxin and little or no difference in the results when compared to placebo. 25
- Lenalidomide
It is a thalidomide derivative with reduced toxicity potential. The high plasma levels of pre- and anti-inflammatory cytokines suggest that the use of non-steroidal immunomodulatory agents contributes to the reduction of symptoms of the syndrome. However, like other drugs mentioned, there appears to be no significant benefit when compared to the use of placebo. 26
- Immunoglobulin
Used intravenously, it points to evidence of reducing peripheral and central neuroimmune activation, acting positively in reducing pain in CRPS when compared to placebo. 27
- Isosorbide dinitrate (ISDN)
This medication determines endothelium-derived vasodilation, which would benefit the changes in microcirculation and decrease in limb temperature that occur in chronic CRPS. Groeneweg et al. 28 cited no difference when compared to placebo.
- Other pharmacological agents
Sahin et al. 29 reported the use of gabapentin in neuropathic pain compared to placebo. Although its anticonvulsant action interferes with analgesia, there was no significant difference in the condition of patients in the two groups. Those who reported improved pain control also experienced more adverse events, such as dizziness, drowsiness and lethargy. Tadalafil is a vasodilator, which acts to inhibit phosphodiesterase. 6 It is used in CRPS to resolve changes in chronic microcirculation that lead to metabolic acidosis and tissue hypoxia. Groeneweg et al., 28 reported a large reduction in pain in the tadalafia group compared to placebo, but without improvement in other markers such as improvement in the sensation of changes in limb temperature and muscle strength.

#### Neuraxial Therapy

Alpha 2 adrenergic agonists administered into the epidural space can reduce pain by decreasing sympathetic system activity. Rauck et al. 30 also mentioned clonidine applied epidurally, associated with adenosine used intrathecally. The improvement in pain seemed to be similar to the use of placebo, and the association with adenosine led to inconvenient arterial hypotension after its application.

Baclofen, an alpha-aminobutyric acid type b receptor agonist (inhibitor of sensory input to the spinal cord) is indicated in cases of irresponsive dystonia. Authors report improvement in dystonia and discuss its intrathecal infusion, whether slowly or quickly. More adverse effects such as headache, nausea and chorea are associated with rapid infusion. Glycine, a glycinergic neurotransmitter, acts to inhibit the process of motor and sensory information, also being useful in the treatment of dystonias. Corticosteroids, such as methylprednisolone, were used intrathecally to treat the syndrome, however, they differed little from the results with intravenous use. 14

#### Stimulation of the Medulla and Dorsal Ganglion Branch

Performed by implanting an epidural electrode in the root that innervates the painful area. The electrical current induces the inhibition of the hyper-excited central neural circuit, a decrease in the sympathetic efferent response and the activation of vasoactive substances. Deer et al. 31 compared stimulation of the spinal cord with stimulation of the dorsal ganglion, with the second method resulting in better results in the pain assessment score. Although there was no difference in patient satisfaction and incidence of adverse events in the two groups studied. Kemler et al., 32 reported good results in the use of stimulation associated with physiotherapy in the first 2 years when compared to the physiotherapy group alone. There is a hypothesis of neuronal adaptation with loss of long-term analgesic effect. 32 33

#### Regional EV Block and Peripheral Sympathetic Block

Although intravenous regional block is a popular practice in the treatment of CRPS, Tran et al., 14 reviewed eleven clinical trials using different substances in the blockade, such as guanethidine, reserpine, droperidol, ketanserin, atropine, lidocaine associated with methylprednisolone, and found no consistent benefit in this recommendation. Other authors also reported this same evidence. 14 34 35

Peripheral sympathetic blockade, of the thoracic stellate ganglion, aims at chemical neurolysis through radiofrequency or the use of anesthetic substances. Several authors have presented more satisfactory results than intravenous blockade. 36 37 38 39

#### Adjuvant Therapy

Physiotherapy, occupational therapy, aerobic exercise, transcutaneous electrical nerve stimulation (TENS) and mirror therapy are the adjuvant procedures most cited in the literature for the treatment of CRPS. Several physical therapy protocols using stretching exercises, muscle strengthening, associated or not with aerobic exercises, edema drainage, temperature contrast baths, ice, heat and TENS, have been reported in series of clinical cases with and without comparison between them. The latter would promote vasodilation and release of endogenous opioids, tending to reduce edema, pain and showing functional benefits when associated with physiotherapy. 40 Although results are promising in some studies, there is no evidence of significant improvement with these methods, especially if used alone. 14 41 42 43 44

Mirror therapy proposes the recognition of the laterality of the hand through constant training in observing the reflection of the unaffected limb in the mirror. According to Moseley et al. 45 constant visualization of the reflex simulating the possibility of using the affected hand would have a positive impact on improving pain and function. Other authors also reported favorable results, with significant reduction in pain, allodynia and improvement in global hand function. 44 46 47 48

## Final Considerations

Complex Regional Pain Syndrome is considered a complicated pathology. Commonly manifested in scientific literature by studies of clinical series with small samples. Patients diagnosed early with the syndrome, between 6 and 18 months of clinical evolution, respond to treatment differently from those with persistent symptoms. The evolution of the condition can be modified through rapid and specific measures, such as the use of specific medications, nerve blocks and adjuvant therapies that will alter the natural history of CRPS. It is estimated that 15% of patients diagnosed at the onset of signs and symptoms of the syndrome will fail to recover satisfactorily. Early observation of a cold extremity tends to mean a poor prognosis. 6

It is emphasized that the available treatment methods help control symptoms and not specifically cure the syndrome. The constant use of medications leads to analgesic tolerance, developed in most patients with CRPS. Also consider the persistence of side effects due to prolonged use of drugs used during treatment. An individualized and coherent therapeutic plan must be established, considering cost and including different classes of medications and procedures that meet other possible needs of the patient. As an example, use of effective tricyclic antidepressants for neuropathic pain and to control anxiety, depression and insomnia. 6 It is suggested that a daily supplementation of 500-1000 mg of Vitamin C can reduce the appearance of CRPS type I in upper/lower extremity trauma and orthopedic surgery. 19

Self-awareness and patient education in the face of a condition compatible with CRPS can lead to the possibility of autonomy in the early use of measures and medications when faced with daily situations and events that could trigger symptom exacerbation. The establishment of reasonable goals during the course of the disease aims to objectively measure symptom improvement or worsening, leading to consequent adjustments in therapeutic measures. This should be developed collaboratively among a multidisciplinary team, the patient, and their family, and it is essential for the quality of treatment. It is crucial to empathetically clarify that the therapeutic approach aims to assist in symptom control. Currently, there is no proven cure for the syndrome, nor are there measures that can alleviate symptoms in all patients.
