# Supplementary material for: Complex Regional Pain Syndrome
Source: Rev Bras Ortop (Sao Paulo). 2024 Apr 22;59(4):e497–503. doi: 10.1055/s-0044-1779331 (PMC11374411; doi:10.1055/s-0044-1779331)
Supplement: Related texts

# Research article: Síndrome da dor complexa regional

**Keywords:** dor crônica, hiperalgesia, síndromes da dor regional complexa

## Abstract

A Síndrome da Dor Complexa Regional é caracterizada por dor regional desproporcional ao evento desencadeante, sem distribuição para dermátomos, com tendência a cronicidade e disfunção. Esta revisão narrativa propõe a atualização de critérios para o diagnóstico e condução da síndrome explicitando dados da epidemiologia, etiologia, fisiopatologia. Baseamo-nos em revisões sistemáticas, narrativas e diretrizes publicadas nos últimos anos, procurando facilitar a suspeição diagnóstica e proporcionando visão ampla de possibilidades terapêuticas.

## Introdução

A Síndrome da Dor Complexa Regional (SDCR) é uma síndrome que representa grande desafio ao diagnóstico e especialmente a condução, dificultada por inúmeras intervenções com pouca fundamentação e evidência científica. É caracterizada por cronicidade e piora progressiva de sintomas que podem determinar incapacidade funcional significativa aos pacientes. O diagnóstico é baseado na história conduzida de forma cautelosa e principalmente, no exame físico criterioso. 1 Em geral, diferencia-se de outras condições que determinam dor crônica por apresentar proeminente alteração autonômica e inflamatória em um segmento ou região, sem seguir dermátomos. 2 3 Mais comum em membros superiores do que inferiores, tem acometimento unilateral com maior frequência. 4 Estudos laboratoriais são irrelevantes, embora úteis ao diagnóstico diferencial em certas circunstâncias. Radiografias podem mostrar sinais de osteopenia regional. Diagnóstico e tratamento precoces são fundamentais para minimizar perda permanente da função do membro.

A partir do início dos anos 1990, o termo Síndrome da Dor Complexa Regional foi adotado ao que antes recebia termos como, distrofia simpático reflexa. 5 A Associação Internacional para o Estudo da Dor (IASP) passou a estabelecer parâmetros, conhecidos como critérios de Budapeste que buscam auxiliar no diagnóstico e categorização do quadro clínico ( Quadro 1 ). 6 Esses parâmetros vêm sendo validados em diretrizes buscando maior especificidade, uma vez que os critérios clínicos úteis podem diferir não somente entre os pacientes, mas, ao longo do tempo da evolução em um mesmo indivíduo.

**Quadro 1:** Critérios de dor para a Síndrome da Dor Complexa Regional (SDCR) revisados e adotados pela IASP (International Association for the Study of Pain) em 2012

**Características gerais:**
A SDCR é caracterizada por dor regional contínua (espontânea e/ou evocada) aparentemente desproporcional em tempo ou grau ao curso habitual de qualquer trauma ou outra lesão conhecida. A dor é regional (não apenas em um território nervoso ou dermátomo específico) e geralmente, os achados anormais sensoriais, motores, vasomotores e/ou tróficos predominam na parte mais distal do membro. A síndrome mostra progressão variável ao longo do tempo.
**Critérios Clínicos para Diagnóstico da SDCR**
1. Dor contínua desproporcional a qualquer evento incitante 2. Deve relatar ao menos um sintoma em três das quatro categorias a seguir: Sensorial : Relatos de hiperalgesia e/ou alodinia Vasomotor : Relatos de assimetria de temperatura e/ou alteração da cor da pele e/ou assimetria da cor da pele Sudomotor/Edema : Relatos de edema e/ou alteração na sudorese e/ou assimetria na sudorese Motor/Trófico : Relatos de diminuição da amplitude de movimento e/ou disfunção motora (fraqueza, tremor, distonia) e/ou alterações tróficas (cabelo, unha, pele) 3. Deve apresentar no momento da avaliação pelo menos um sinal* em duas ou mais das seguintes categorias: Sensorial : Evidência de hiperalgesia (à alfinetada) e/ou alodinia (ao toque leve e/ou pressão somática profunda e/ou movimento articular) Vasomotor : Evidência de assimetria de temperatura e/ou alterações e/ou assimetria da cor da pele Sudomotor/Edema : Evidência de edema e/ou alterações de sudorese e/ou assimetria de sudorese Motor/Trófico : Evidência de diminuição da amplitude de movimento e/ou disfunção motora (fraqueza, tremor, distonia) e/ou alterações tróficas (cabelo, unha, pele) * Um sinal é contado apenas se for observado no momento do diagnóstico. 4. Não há outro diagnóstico que explique melhor os sinais e sintomas

Tradução em português baseada em Diretrizes de Diagnóstico e Tratamento da SDCR. Harden RN, McCabe CS, Goebel A, et al. Complex regional pain syndrome: practical diagnostic and treatment guidelines, 5th edition. Pain Med 2022;23(Suppl 1):S1-S53. 6

## Tipos de SDCR

Classicamente a SDCR é apresentada em três subtipos. No tipo I, inexiste comprometimento específico de nervo periférico, evidenciado no exame clínico ou eletrofisiológico, síndrome relativamente limitada na qual predominam os sinais vasomotores. No tipo II, denominada anteriormente causalgia, há envolvimento de lesão nervosa identificável determinante da dor neuropática, uma síndrome relativamente limitada na qual predominam a dor neuropática/anormalidades sensoriais. Alguns autores referem um terceiro tipo que atende parcialmente aos critérios, porém, não são especificados nos outros dois tipos, define-se o tipo III como uma SDCR bastante florida, embora sinais de alteração motora/trófica tenham sido observados em todos os três grupos, eles dobraram em frequência entre os pacientes do Subgrupo 3. 3 4 7 Afora os subtipos I, II e III citados anteriormente, encontramos referência a diferenciação clínica da síndrome em padrão “quente” e “frio”, respectivamente caracterizado por extremidade quente, edemaciada, seca, avermelhada e outra por extremidade fria, menos edemaciada, com suor, azulada. Harden et al, reportaram que o padrão mais comum é o “quente”, sendo que esse apresenta menor tempo de duração da síndrome comparado ao padrão “frio”. 7

## Epidemiologia

A Síndrome da Dor Complexa Regional (SDCR) engloba frequência de 5,46-26,2 a cada 100.000 pessoas por ano. 6 8 Mulheres são três vezes mais afetadas que homens. A idade mais prevalente varia entre 46 e 53 anos. Membros superiores são mais envolvidos que os inferiores. Shim et al., 5 referiram que haverá maior risco para o desenvolvimento da SDCR em mulheres que sofrerem traumas de alta energia no membro superior. Bruehl, 2 relata taxas de incidência diferentes para os tipos I e II da síndrome, respectivamente 5,46 por 100 mil pessoas ao ano e 0,82 por 100 mil pessoas ao ano.

## Etiologia e Fisiopatologia

Fraturas são referidas como o principal fator precipitante da SDCR em membros superiores e inferiores, sendo a fratura de radio e ulna em sua extremidade distal a região mais acometida pela síndrome. 1 SDCR é mais provável de ocorrer após fraturas do rádio distal em pacientes tratados com gessos apertados ou após um evento nociceptivo ou neuropático, como compressão do nervo mediano, hiperdistração, instabilidade da articulação radioulnar distal ou fratura da ulna. 1 Outros traumas, cirurgias e mesmo condições inflamatórias podem ser fatores manifestadores. Urits et al., 3 mencionaram que em 7% dos casos há desenvolvimento da síndrome do tipo I, usualmente nas 8 semanas seguintes ao evento traumático. Relevante observar, persistência de dor e edema após as primeiras semanas do trauma como sinais para o aparecimento da síndrome. Há relato de incidência pouco maior que 8% em pacientes submetidos a liberação do túnel do carpo. 3 Entretanto, em revisão sistemática recente, que buscou evidências disponíveis sobre ocorrência da SDCR após a liberação cirúrgica do túnel do carpo, nosso grupo encontrou incidência de 2 a 5%. 8 E, listou como fatores de risco para o aparecimento da síndrome após cirurgia, sexo feminino, idade a partir da quinta década, cirurgia na mão dominante, tempo de torniquete e uso de imobilização após procedimento cirúrgico. 8

A fisiopatologia da SDCR é incerta. O mecanismo exato que determina início e manutenção da dor permanece desconhecido. 3 As teorias tendem ao motivo pelo qual a dor persiste e não ao modo como surge. Há hipótese de que neuropeptideos ligados ao gene de moduladores químicos, como a calcitonina, incitem inflamação neurogênica e sensibilização de catecolaminas. A natureza persistente da dor na SDCR aparenta estar conectada a características autonômicas do sistema nervoso simpático como a regulação positiva de adrenoceptores e diminuição da densidade de fibras nervosas cutâneas. 9 A dor patológica parece ser provinda de alterações no processamento sensorial e motor, ou seja, função motora, feedback sensorial, representação corporal, propriocepção e integração sensório-motora. No entanto, há evidências muito limitadas que modificações desse processamento levariam realmente a cronicidade da dor. 10 A teoria multifatorial é mais aceita e inclui aspectos que interferem desde o momento do evento agressor como sensibilização do sistema nervoso, disfunção autonômica, alterações inflamatórias, além de possível relação com fatores psicológicos e predisposição genética. 5 Também, tabagismo foi apontado como fator de risco significativo ao desenvolvimento da SDCR. 7

As disfunções ocasionadas pela SDCR podem incluir perda de mecanismos normais do fluxo arteriovenoso e alterações definitivas de respostas neurológicas centrais, resultando em isquemia segmentar e morte celular. 10 Esses por sua vez, determinam dor, edema, rigidez articular e atrofia do segmento. Em síntese, a síndrome da dor complexa regional traz em sua essência exagero ou prolongamento anormal de eventos fisiopatológicos esperados após determinado episódio traumático, alterando a percepção e submetendo o indivíduo a uma interação complexa de fatores fisiológicos e psicológicos. 10

## Diagnóstico

A suspeição do desenvolvimento da SDCR inicia no aparecimento de dor exacerbada, normalmente não esperada após trauma ou cirurgia. Outros fatores devem ser considerados como, manutenção imprevista de edema, recuperação mais lenta que habitual, transtornos do sono e ausência de melhora da dor com analgésicos comuns. 11 O diagnóstico pode ser auxiliado com o uso dos critérios de Budapeste ( Quadro 1 ). 6

Distingue-se de outras síndromes dolorosas por presença de disfunção autonômica, alterações inflamatórias regionais persistentes e ausência de distribuição seguindo dermátomos. 5 Pacientes descrevem a dor como dilacerante ou em queimação, muitas vezes exacerbada pelo frio. Além da dificuldade no sono, também referem ansiedade e inquietude. Comumente encontram-se no exame físico, alodinia (resposta dolorosa a estímulo habitualmente não doloroso), hiperalgesia, hiperpatia associadas ao edema. Poderão estar presentes alterações tróficas provenientes da disfunção autonômica como sudorese anormal, mudança de temperatura e textura da pele. Identifica-se a extremidade envolvida “quente” e edemaciada ou “fria” e atrófica. Com o passar do tempo, dor e edema levam a rigidez articular, especialmente de dedos, punhos e ombros. 10

Crianças e adolescentes podem ser acometidos pela SDCR. É mais frequente no membro inferior de meninas adolescentes, próximo aos 12 anos de idade, que tiveram evento traumático na região de tornozelo e pé. 1 O quadro clínico inclui dor, alteração autonômica e sensibilidade exacerbada aos estímulos dolorosos e mesmo não habitualmente dolorosos (alodinia). Como em adultos, pode evoluir para distúrbios motores e alterações tróficas. O prognóstico tende a ser melhor se comparado a ocorrência em adultos. 12

Diante de quadro álgico intenso que tende a cronicidade, torna-se indispensável afastar outras patologias na ausência de achados físicos objetivos, como alterações tróficas, edema e osteopenia. Dor e incapacidade ligados a SDCR apontam para comorbidades psicológicas que criam ciclo vicioso de dor, isolamento e depressão. 4

Piñal (Enzine, 2019), em seu verdadeiro desabafo publicado na revista da IFSSH (International Federation of Societies for Surgery of the Hand) sobre o tema, coloca em dúvida a existência de tipos da síndrome e expõe a controvérsia no diagnóstico e tratamento. Refere a teoria da amplificação do estímulo em um nervo que está sendo “irritado”, mesmo sem uma lesão mecânica estabelecida, o que resultaria em sintomas mais abrangentes no membro acometido. O autor publicou recentemente estudo de 53 pacientes, média de 55 anos de idade, diagnosticados anteriormente como portadores de SDCR, que apresentavam sintomas e sinais atípicos de dor e disestesias em membro superior. Enfatizou a incapacidade dos indivíduos em fletir completamente os dedos (preensão), e, demonstrou melhora dos sintomas após liberação do ligamento transverso do carpo. Apenas 6 dos 53 pacientes mantiveram o quadro prévio a cirurgia, avaliados com escala analógica visual da dor e questionário DASH (Disabilities of the Arm, Shoulder and Hand Questionnaire). 13 Nessa linha de fundamentação, Chang et al. 4 discutiram os efeitos psicossociais criadores e rotuladores de síndromes. Partiram da premissa que a SDCR pode apresentar conjunto de sintomas inconsistentes e vagos, facilmente atendidos no quadro de outros diagnósticos. Citaram exemplos de artigos que confirmaram altas porcentagens da síndrome após determinado procedimento cirúrgico sem que os pacientes sequer completassem sinais e sintomas como os citados nos critérios apresentados no quadro. 2 Ressaltaram a importância do minucioso exame clínico para identificar possíveis origens da dor e evitar conduções incorretas que poderão trazer consequências desastrosas ao paciente.

Exames complementares de imagem e laboratoriais geralmente estarão inalterados e pouco auxiliarão no estabelecimento do diagnóstico da SDCR. Após duas semanas ou mais do início do quadro, radiografias poderão apresentar sinais de osteopenia e reabsorção óssea, principalmente subcondral e periarticular. Estima-se que até 30% dos pacientes não apresentarão anormalidades radiográficas no momento do diagnóstico da síndrome. 10 Embora termografia, cintilografia, eletroneuromiografia e ultrassonografia possam demonstrar alterações na vigência da SDCR, em última análise, não há evidência científica para sua utilização indiscriminada no diagnóstico da síndrome. 5

## Tratamento

A dificuldade para proposição de tratamento na SDCR vai no mesmo sentido do estabelecimento de seu diagnóstico. Numerosas terapias estão sugeridas em series de casos isoladas ou em comparações, revisões narrativas, diretrizes e dados agrupados em revisões sistemáticas e tentativas de metanálise. Na procura simples em uma das plataformas de busca científica é possível captar mais de quatro mil artigos publicados entre os anos 1945 e 2022, sendo a maioria após o ano 2000. Encontram-se as primeiras publicações com os nomes de causalgia, dor reflexa, distrofia simpático reflexa, distrofia de Sudeck, que exprimem as mesmas dificuldades encontradas nos trabalhos mais recentes, ou seja, a discussão no estabelecimento de diagnóstico e conduta. Isso nos leva a refletir, estamos estudando a causa desta suposta síndrome tentando descobrir sua prevenção ou expondo incontáveis opções de tratamento para sintomas mal reconhecidos ou mal diagnosticados.

Pacientes com SDCR tipo 2 têm uma lesão de nervo periférico identificável ou documentada. As intervenções cirúrgicas que corrigem o dano do nervo ou protegem o nervo podem diminuir a incidência de focos nociceptivos, diminuir os sintomas e melhorar a função. Da mesma forma, os pacientes com SDCR tipo 1 podem ter um foco nociceptivo não neural ou mecânico, que, se corrigido, facilitará a recuperação da extremidade. 1

Utilização de numerosos fármacos, terapia neuraxial, estimulação de medula e ramo do gânglio dorsal, bloqueio regional endovenoso e simpático periférico, terapia adjuvante, entre outros, são citados no tratamento da SDCR de forma isolada ou conjunta. 6 14 Para expor as opções de tratamento atual da SDCR nos baseamos em revisões sistemáticas, narrativas e diretrizes dos últimos anos. Procuramos organizar em tópicos para facilitar o entendimento diante da superabundância de possibilidades. Porém, os estudos sugerem que ensaios controlados randomizados de alta qualidade ainda são necessários para realizar recomendações sólidas quando ao tratamento da SDCR. 1

## Fármacos

- Bifosfonados
Podendo ser administrados via oral ou endovenosa; buscam inibir a reabsorção óssea que acompanha a SDCR, Pesquisas afirmam que os bifosfonados apresentam claros benefícios no tratamento da síndrome. 14 15 Há relatos de melhora significativa de alodínea, hiperalgia, edema e mobilidade articular. As infusões de pamidronato são bem toleradas e fáceis de administrar. Vários pacientes relataram reações menores no local da infusão e sintomas leves que se resolveram dentro de 6 a 24 horas após administração. O mecanismo do efeito analgésico dos bifosfonados permanece especulativo. Eles são potentes inibidores da atividade osteoclástica, podem desempenhar papel na modificação de citocinas inflamatórias (como interleucina-1) e outros fatores sistêmicos (como prostaglandina E2) envolvidos na sensibilização de nociceptores dolorosos e mecanorreceptores de baixo limiar. 15
- Corticóides
Christensen et al. 16 compararam prednisona oral e placebo no tratamento da SDCR e obtiveram melhora de dor, edema, suor palmar, habilidade de pinça e dedilhar com o uso do corticoide. Pois estes mediam a inibição de inflamação e a analgesia. Também, outros autores relataram melhor controle de dor e edema com uso de prednisona oral quando comparada ao antinflamatório não hormonal. 17
- Calcitonina
Quando comparada a placebo ou outras terapias, como fisioterapia, apresentou benefícios insignificantes no tratamento da SDCR. 14 Acreditando que sua ação determinaria inibição da reabsorção óssea e analgesia mediada por beta endorfina.
- Antinflamatório não hormonais
Breuer et al. 18 não encontraram diferença significativa quanto a melhora de dor e edema, em pacientes diagnosticados com a síndrome, quando compararam uso de parecoxib endovenoso e solução fisiológica. Seu uso foi avultado pela função de promover a inibição da COX2 tendendo a reduzir a sensibilização periférica e normalizar o limiar da dor a pressão local.
- Vitamina C
A revisão sistemática realizada por Giusta et al., 19 avaliou a eficácia da vitamina C na prevenção da SDCR tipo I em fraturas ou cirurgias das extremidades superiores e inferiores. Cinco dos seis estudos analisados eram favoráveis ao uso profilático da dose diária de 500-1000 mg de vitamina C por 45-50 dias após cuidados ortopédicos ou traumatológicos para prevenção da SDCR tipo I. Apenas um estudo não encontrou algum benefício na suplementação de vitamina C em comparação com placebo na prevenção da SDCR. Portanto, nessa análise da literatura sugere-se a suplementação diária de 500-1000 mg de VC pode reduzir o aparecimento de SDCR tipo I em trauma de extremidades superiores/inferiores e em cirurgia ortopédica.
- Antagonista do receptor N-Methyl-D-Aspartato (NMDA)
Devido à sua ação nos mecanismos de sensibilização periférica e central, especificamente na cascata de sensibilização neuronal, que leva a ativação e liberação de substâncias no sistema nervoso central (SNC) como o glutamato, aumentando a eficiência da transmissão sináptica do sinal de dor. No entanto, alguns artigos referiram não haver melhora significativa com o uso de antagonistas do NMDA ou de placebo. 18 20 21
A Ketamina age no SNC como bloqueadora de receptores, não competitiva. Autores referem diminuição de alodínea/hiperalgia com uso tópico de ketamina comparado ao placebo. 22 Bons resultados foram também referidos com o uso endovenoso. Atenção deve ser observada para a curta duração do tratamento endovenoso, evitando sintomas indesejáveis como náuseas, vômitos e efeitos psicomiméticos. 23 24
- Toxina botulinica A
Como a toxina botulínica tende a diminuir a sensibilização periférica e a percepção de dor central ao inibir a neurotransmissão da dor tanto superficial como profunda e bloquear o transporte axonal retrógrado, sua ação foi avaliada no tratamento da SDCR. Porém há referência de dor intensa durante a aplicação da toxina e pouca ou nenhuma diferença nos resultados quando confrontados ao placebo. 25
- Lenalidomida
É um derivado da talidomida com potencial de toxicidade reduzido. Os níveis plasmáticos elevados de citocinas pré e antinflamatórias sugerem que o uso de agentes imunomoduladores não esteróides contribuem na diminuição de sintomas da síndrome. Porém, como outros fármacos citados, parece não haver benefício significativo quando comparado ao uso de placebo. 26
- Imunoglobulina
Usada de forma endovenosa, aponta para evidências na redução da ativação neuroimune periférica e central, agindo positivamente na diminuição da dor na SDCR quando comparada ao placebo. 27
- Isossorbida dinitrato (ISDN)
Esta medicação determina vasodilatação derivada do endotélio o que beneficiaria as alterações na microcirculação e diminuição de temperatura do membro que ocorrem na SDCR crônica. Groeneweg et al., 28 citaram não haver diferença quando confrontada ao placebo.
- Outros agentes farmacológicos
Sahin et al., 29 reportaram o uso de gabapentina na dor neuropática comparando-a ao placebo. Apesar de sua ação anticonvulsivante interferir na analgesia não houve diferença significativa no quadro de pacientes nos dois grupos. Os que reportaram melhora no controle da dor, também apresentaram mais eventos adversos, como tontura, sonolência e letargia. A Tadalafila é vasodilatador, que atua na inibição da fosfodiesterase. 6 É utilizada na SDCR para dirimir a alteração da microcirculação crônica que leva a acidose metabólica e a hipóxia tecidual. Groeneweg et al., 28 reportaram grande redução da dor no grupo da tadalafia comparado ao placebo, porém sem melhora de outros marcadores como melhora na sensação de alteração de temperatura do membro e de força muscular.

## Terapia Neuraxial

Agonistas alfa 2 adrenérgico administrados no espaço epidural podem reduzir a dor diminuindo a atividade do sistema simpático. Rauck et al., 30 citaram a clonidina aplicada epidural, também, associada a adenosina utilizada intratecal. A melhora da dor pareceu ser semelhante ao uso de placebo, sendo que a associação com adenosina trouxe a inconveniente hipotensão arterial após sua aplicação.

O Baclofeno, agonista do receptor do ácido alfa-aminobutírico tipo b (inibidor da entrada sensorial na medula) é indicado em casos de distonia irresponsiva. Autores referem melhora no quadro de distonia e discutem sua infusão intratecal, se de forma lenta ou rápida. Mais efeitos adversos como cefaleia, náusea e coreia são associados a infusão rápida. A glicina, neurotransmissor glicinérgico, age na inibição do processo de informação motora e sensorial, também sendo útil no tratamento das distonias. Corticoides, como a metilprednisolona, foram utilizados intratecal no tratamento da síndrome, porém, pouco diferiram do resultado com o uso endovenoso. 14

## Estimulação da Medula e Ramo do Gânglio Dorsal

Realizada por meio do implante de eletrodo epidural na raiz que inerva a área dolorida. A corrente elétrica induz a inibição do circuito neural central hiper excitado, diminuição da resposta eferente simpática e ativação de substâncias vasoativas. Deer et al., 31 compararam estimulação da medula com estimulação do gânglio dorsal, resultando o segundo método em melhor resultado no escore de avaliação da dor. Embora não haver diferença na satisfação dos pacientes e incidência de eventos adversos nos dois grupos estudados. Kemler et al., 32 reportaram bons resultados na utilização da estimulação associada a fisioterapia nos primeiros 2 anos quando comparada ao grupo de fisioterapia isolado. Há hipótese de adaptação neuronal com perda do efeito analgésico a longo prazo. 32 33

## Bloqueio Regional EV e Simpático Periférico

Embora o bloqueio regional endovenoso seja prática popular no tratamento da SDCR Tran et al., 14 revisaram onze ensaios clínicos com uso de diversas substâncias no bloqueio, como, guanetidina, reserpina, droperidol, ketanserina, atropina, lidocaína associada a metilprednisolona e não encontraram benefício consistente nessa indicação. Outros autores também reportaram essa mesma evidência. 14 34 35

O bloqueio simpático periférico, do gânglio estrelado torácico, visa a neurolise química por radiofrequência ou uso de substâncias anestésicas. Vários autores apresentaram resultados mais satisfatórios do que o bloqueio endovenoso. 36 37 38 39

## Terapia Adjuvante

Fisioterapia, terapia ocupacional, prática de exercícios aeróbicos, estimulação nervosa elétrica transcutânea (TENS) e terapia do espelho são as condutas adjuvantes mais citadas na literatura para o tratamento da SDCR. Diversos protocolos de terapias físicas utilizando exercícios de alongamento, fortalecimento muscular, associados ou não a exercícios aeróbicos, drenagem de edema, banhos com contraste de temperatura, gelo, calor e TENS (estimulação nervosa elétrica transcutânea), foram reportados em séries de casos clínicos com e sem comparação entre eles. Esse último, promoveria vasodilatação e liberação de opióides endógenos, tendendo a reduzir edema, dor e mostrando benefícios funcionais quando associado a fisioterapia. 40 Embora os resultados sejam promissores em alguns estudos, não há evidência de melhora significativa com esses métodos, especialmente se usados de forma isolada. 14 41 42 43 44

A terapia do espelho propõe o reconhecimento da lateralidade da mão no treinamento constante da observação do reflexo do membro não afetado no espelho. Segundo Moseley et al. 45 a visualização constante do reflexo simulando a possibilidade de uso da mão afetada impactaria positivamente na melhora da dor e função. Outros autores também referiram resultados favoráveis, com redução significativa da dor, alodínea e melhora da função global da mão. 44 46 47 48

## Considerações Finais

A Síndrome da Dor Complexa Regional é considerada patologia complicada. Comumente, manifestada na literatura científica por estudos de séries clínicas com amostras pequenas. Pacientes diagnosticados precocemente com a síndrome, entre 6 e 18 meses de evolução clínica, respondem ao tratamento de maneira diferente daqueles com sintomas persistentes. A evolução do quadro pode ser modificada diante de condutas céleres e pontuais, como uso de medicamentos específicos, bloqueios nervosos e terapias adjuvantes que alterarão a história natural da SDCR. Estima-se que 15% dos pacientes diagnosticados no início de sinais e sintomas da síndrome falharão em recuperar satisfatoriamente. A observação antecipada de uma extremidade fria tende a significar mau prognóstico. 6

Enfatiza-se que os métodos de tratamento disponíveis auxiliam no controle de sintomas e não especificamente na cura da síndrome. O uso constante de medicamentos leva a tolerância analgésica, desenvolvida na maioria dos pacientes com SDCR. Também considerar, a persistência de efeitos colaterais por uso prolongado de drogas utilizadas durante o tratamento. Um plano terapêutico individualizado e coerente deve ser estabelecido, considerando custo e incluindo diferentes classes de medicamentos e procedimentos que venham atender outras eventuais necessidades do paciente. Como exemplo, uso de antidepressivos tricíclicos eficazes para dor neuropática e para controle de ansiedade, depressão e insônia. 6 Sugere-se que uma suplementação diária de 500-1000 mg de Vitamina C pode reduzir o aparecimento de SDCR tipo I em trauma de extremidades superiores/inferiores e em cirurgia ortopédica. 19

Autoconhecimento e conscientização do paciente diante de quadro compatível com SDCR, leva a possível autonomia para uso antecipado de medidas e medicamentos diante de situações e acontecimentos diários que possam engatilhar a piora de sintomas. O estabelecimento de metas razoáveis durante a condução da doença visa mensurar objetivamente, melhora ou piora de sintomas e consequente alteração na aplicação de medidas terapêuticas. Deve ser construído em conjunto, entre equipe multidisciplinar, paciente, familiares e é primordial para qualidade do tratamento. Imprescindível, esclarecer de forma empática que a proposta terapêutica visa auxiliar no controle de sintomas. Atualmente, não existe tratamento comprovado para a cura da síndrome, ou mesmo, medidas que venham a dirimir os sintomas em todos os pacientes.
